# Vaccinating women previously treated for human papillomavirus-related cervical precancerous lesions is highly cost-effective in China

**DOI:** 10.3389/fimmu.2023.1119566

**Published:** 2023-03-27

**Authors:** Maosheng Zou, Hanting Liu, Huan Liu, Mengjie Wang, Zhuoru Zou, Lei Zhang

**Affiliations:** ^1^ China–Australia Joint Research Centre for Infectious Diseases, School of Public Health, Xi’an Jiaotong University Health Science Centre, Xi’an, Shaanxi, China; ^2^ Melbourne Sexual Health Centre, Alfred Health, Melbourne, VIC, Australia; ^3^ Central Clinical School, Faculty of Medicine, Monash University, Melbourne, VIC, Australia

**Keywords:** health economics (cost-effectiveness analysis), HPV vaccination, cervical precancerous lesions, women, healthcare provider

## Abstract

**Background:**

The 2021 Chinese Expert Consensus on the Clinical Application of the Human Papillomavirus (HPV) Vaccine recommended vaccination for women who previously received ablative or excisional treatment for high-grade squamous intraepithelial lesion (HSIL). This study evaluates the cost-effectiveness of HPV vaccination in women previously treated for cervical precancerous lesions.

**Methods:**

We used a Markov model to simulate the disease progression of both low- and high-risk HPV subtypes. We followed a cohort of 100,000 women aged 18-45 years who received treatment for cervical precancerous lesions for a lifetime (80 years). We used the Incremental Cost-Effectiveness Ratios (ICER) with a 5% discount rate to measure the cost-effectiveness of nine vaccination strategies, including a combination of HPV bivalent (HPV-2), quadrivalent (HPV-4) and nonavalent vaccine (HPV-9), each with three vaccination doses (one-, two- and three-dose). We conducted one-way sensitivity analysis and probabilistic sensitivity analysis. We followed the CHEERS 2022 guidelines.

**Results:**

Compared to the status quo, the nine vaccination strategies would result in $3.057-33.124 million incremental cost and 94-1,211 incremental quality-adjusted life-years (QALYs) in 100,000 women previously treated for cervical precancerous lesions. Three vaccination strategies were identified on the cost-effectiveness frontier. In particular, ICER for one-dose HPV-4 vaccination was US$10,025/QALY compared to the status quo (no vaccination); ICER for two-dose HPV-4 vaccination was US$17,641//QALY gained compared to one-dose HPV-4 vaccination; ICER for three-dose HPV-4 vaccination was US$27,785/QALY gained compared with two-dose HPV-4 vaccination. With a willingness-to-pay of three times gross domestic product per capita (US$37655), three-dose HPV-4 vaccination was the most cost-effective vaccination strategy compared with the lower-cost non-dominated strategy on the cost-effectiveness frontier. A probabilistic sensitivity analysis confirmed a 99.1% probability of being cost-effective. If the cost of the HPV-9 is reduced to 50% of the current price, three-dose HPV-9 vaccination would become the most cost-effective strategy.

**Discussion:**

Three-dose HPV-4 vaccination is the most cost-effective vaccination strategy for women treated for precancerous cervical lesions in the Chinese setting.

## 1 Introduction

Cervical cancer is the second most common cancer among women in low- and lower-middle-income countries and is a major challenge for global health (1). According to the International Agency for Research on Cancer (IARC), there were 604,127 new cases and 341,831 deaths of cervical cancer worldwide in 2020, of which 109,741 new cases and 59,060 deaths occurred in China, accounting for 18.1% and 17.3% of new cases and cervical cancer mortality worldwide respectively (2). Moreover, the age-standardized mortality rates of cervical cancer age-standardized mortality rate (ASMR) in urban has approximately increased from 2‰ to 5‰ during 2008-2018, posing a major threat to the health of Chinese women (3).

Vaccination is the primary strategy to prevent HPV infection and hence consequent cervical cancer in women. Available HPV vaccines in China include bivalent (HPV-2), quadrivalent (HPV-4), and nonavalent vaccines (HPV-9). HPV-2 includes the domestically-produced products Cecolin and Vozevir and the imported product Cervarix. Previous studies have shown that there is little difference in safety and efficacy between domestic and imported HPV-2 (4), but the price of domestic HPV-2 is about half as much as imported HPV-2. HPV-4 and HPV-9 provide more comprehensive protection against HPV-related diseases than HPV-2, although they are more expensive. Several studies have confirmed the effectiveness of the HPV vaccine in preventing HPV infection, genital warts, and cytological and histological abnormalities of the cervix (4–9). Moreover, although the HPV-4 selected by our model is a preventive HPV vaccine, a relevant study has found that the HPV-4 after treatment may have a therapeutic effect in women with residual/recurrent CIN 1 or high-grade CIN (CIN 2-3) (10). In addition, some studies have shown that universal HPV vaccination for pre-adolescent girls is significantly cost-effective (11–17). In 2020, the World Health Assembly adopted the Global Strategy for the Elimination of Cervical Cancer, one of the strategic objectives of which is to increase HPV vaccination coverage.

Cervical precancerous lesions often present as cervical intraepithelial neoplasia (CIN1, CIN2, and CIN3) and reflect the potential severity of cervical carcinogenesis (18). Treatment options for cervical precancerous lesions currently include cryotherapy, thermal ablation, loop electrosurgical excision procedure (LEEP), cold knife and hysterectomy, etc (19–22). A review study has shown that the risk of cervical cancer is much higher in women treated for precancerous cervical lesions than in the general population (23). The recurrence rate of highly squamous intraepithelial lesions (HSIL) after conservative local treatment is as high as 5%-10%, and the risk of invasive cancer is 2-4 times higher than that of the general population (24). A retrospective analysis conducted in Korea showed that HPV-4 vaccination in patients with HSIL after LEEP treatment significantly reduced the risk of recurrence of HSIL associated with HPV16/18 (25). In addition, the results of a prospective case-control study and a prospective RCT conducted in Italy in 2018 both showed that HPV-4 vaccination significantly reduced the recurrence rate of HSIL (CIN2+) after treatment of HPV-related diseases such as CIN 2+ (26, 27). Accumulating evidence demonstrates that the HPV vaccine can induce a large number of antibodies in the basal layer of the cervix, which prevents the regenerating tissue from self-infecting and prevents HPV from entering the uninfected basal layer cells, thus preventing the recurrence of HSIL (CIN2+) (25). The 2021 Chinese Expert Consensus on Clinical Application of Human Papilloma Virus Vaccine states that HPV vaccination is recommended for women who have undergone ablative or excisional treatment for previous HSIL (28).

Studies have been conducted to evaluate the cost-effectiveness of HPV vaccination in the Chinese population (11, 29–33), however, these studies have focused on the general population and there are no studies to evaluate the cost-effectiveness of HPV vaccination in women after treatment for cervical precancerous lesions. Therefore, we conducted the present study to inform clinical practice and treatment guidelines for HPV vaccination in women after treatment for cervical precancerous lesions.

## 2 Methods

### 2.1 Model construction

**Figure 1:**
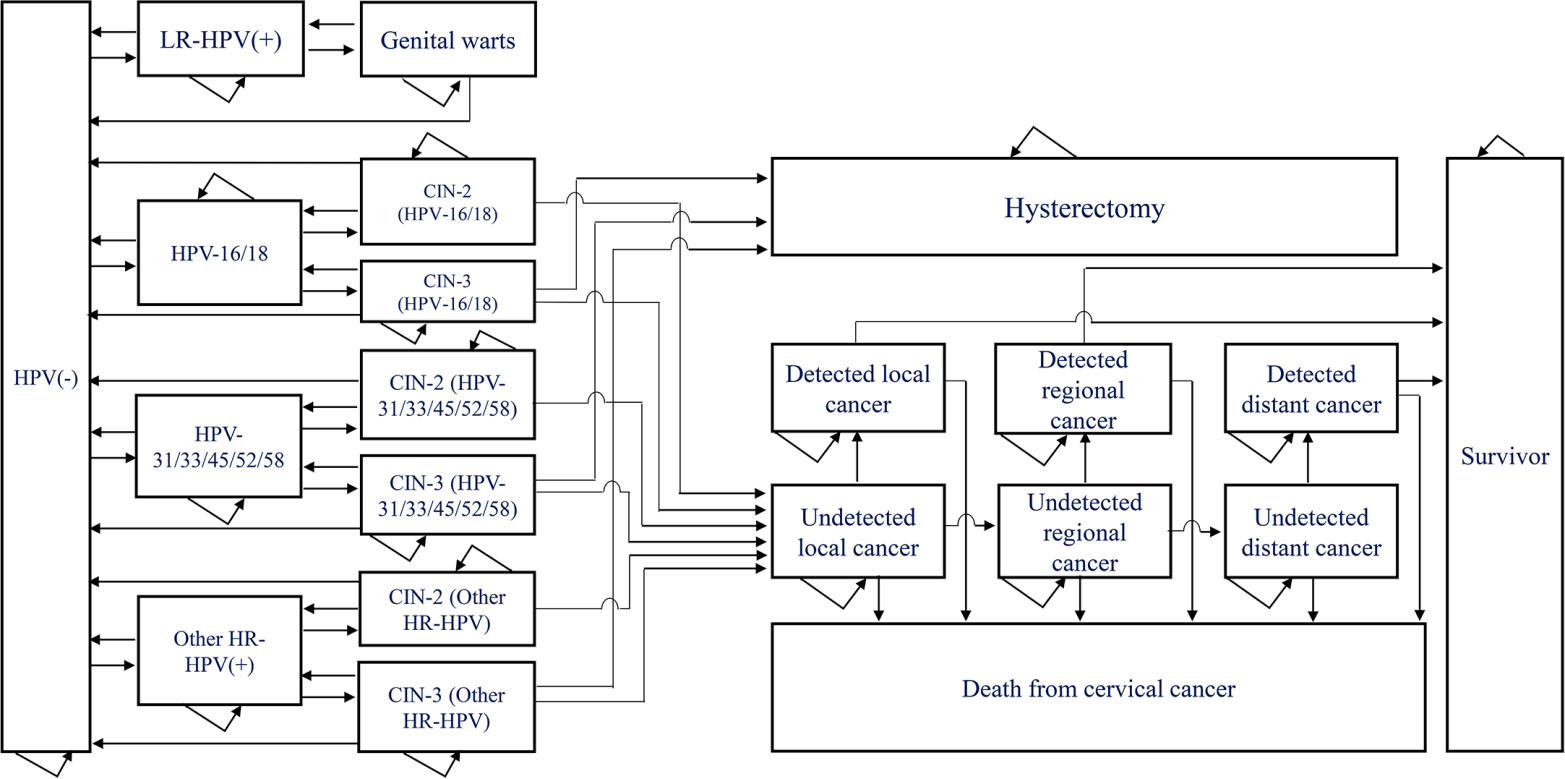
Markov model of the natural history of low and high risk type of HPV. HPV, human papillomavirus; CIN, cervical intraepithelial neoplasia; LR-HPV(+): HPV6/11 only, no other low-risk HPV infections considered; HPV-16/18(+): infected with HPV-16 alone, with HPV-18 alone or with both HPV-16 and 18; HPV-31/33/45/52/58(+): HPV-31, HPV33, HPV45, HPV52 or HPV58 alone, or co-infection with two or more of these five types of HPV; Other HR-HPV(+): Infection with high-risk HPV types other than HPV16, HPV18, HPV-31, HPV33, HPV45, HPV52 or HPV58.

We constructed a Markov model using TreeAge Pro 2021 to simulate the transition from low-risk type HPV infection to genital warts and from high-risk type HPV infection to cervical cancer in 100,000 women aged 18-45 years after treatment for cervical precancerous lesions for a lifetime (life expectancy 80 years) (**Figure 1**) . The Consolidated Health Economic Evaluation Reporting Standards 2022 (CHEERS 2022) is used for this health economic evaluation (34).

The model contained a total of 21 health states, including HPV negative, low-risk HPV positive, genital warts, high-risk HPV positive, CIN2, CIN3, hysterectomy, local cancer, regional cancer, distant cancer, death from cervical cancer, etc. HPV-positive individuals may clear the infection or progress to HPV-related lesions (genital warts or CIN2+), genital warts may regress to HPV negative or low-risk HPV positive, CIN2 and CIN3 may regress to HPV negative, high-risk HPV positive or progress to local cervical cancer, A proportion of CIN3 patients who undergo hysterectomy will no longer be at risk of developing cervical cancer. We set an age-specific natural mortality rate for all health states and an additional rate of death from cervical cancer at each stage for patients with cervical cancer. A proportion of patients diagnosed with cervical cancer were treated and we considered survivors of cervical cancer treated for more than 20 years to be cured. The length of each cycle of the model was 1 year, and we made a half-cycle correction.

### 2.2 Definition of scenarios

The WHO Strategic Advisory Group of Experts on Immunization (SAGE) concluded that single-dose schedules provide comparable efficacy to the two or three-dose regimens. We set up nine different vaccination strategies in the model, including one, two, and three doses of HPV-2, HPV-4, and HPV-9 vaccination. The status quo is no vaccination. In consideration of price and protection, the HPV-2 we have chosen is a domestic vaccine named Cecolin. A study of women aged 27-45 years in China showed that nearly 60% of this age group were willing to receive the HPV vaccine (35), so we assumed that the vaccine coverage was 60%. The vaccine efficacy of each vaccine is derived from the published literature (7, 36–38). We assumed that one-dose, two-dose, and three-dose vaccinations have the same efficacy but the duration of protection varies. Based on the results of the two follow-up studies (39, 40), we estimated the duration of protection for the one-dose vaccination to be approximately 9 years, after which we set the antibody titer at year 9 after the one-dose vaccination as the effective antibody titer threshold for the vaccine and thus estimated the effective duration of protection for the two-dose and three-dose vaccinations, which was 9 years (3.4-14.5) for the one-dose vaccination, 22 years (19.3-26.2) for the two-dose vaccination and 44 years (41.0-47.5) for the three-dose vaccination. We assumed the same duration of protection for all three HPV vaccines and did not consider cross-protection of the vaccines.

### 2.3 Model parameters

We obtained the probability of annual transition from the published literature (**Supplementary Table 1**), and the incidence of HPV infection at each age was fitted to the results of the follow-up studies (**Supplementary Table 2**). Mortality rates from other causes were obtained from the Chinese Yearbook of Health Statistics 2021, while mortality of cervical cancer was obtained from the published literature (13). We conducted this study from a healthcare provider perspective, so we only considered all direct medical costs within the health system, including the cost of vaccination, treatment of genital warts, treatment of cervical cancer, and health care. The cost of vaccination includes the price of the vaccine and vaccination consumables. The price of the vaccine is the winning bid price announced by Shanghai Health Affairs Service Center in 2021 for the HPV-2 (Cecolin, $51.0 per dose), HPV-4 (Gardasil, $123.7 per dose), and HPV-9 (Gardasil 9, $201.2 per dose), and the vaccination service fee is based on the non-immunization vaccination service fee ($3.4 per dose) measured by the Development and Reform Commission of Hainan Province. Through domestic substitution and negotiations, we expect further reductions in vaccine prices in the future. Visual inspection with acetic acid (VIA), Colposcopy, Biopsy, and Papanicolaou test (Pap) was used for the diagnosis of CIN2, CIN3, and cervical cancer. The cost data for these tests are derived from the published literature (13). Cost data for genital wart treatment, cervical cancer treatment, and health care was based on published literature (41–43). Costs were converted to US dollars in 2021 based on the published Consumer Price Index (CPI), the CPI, and gross domestic product (GDP) per capita obtained from the National Statistics Office. Health utility values for each health state in the model were obtained from the published literature (44, 45). The parameters used in the model are shown in **Table 1**.

**Table 1:** Variables used in the model.

	Base-case	Range	Distribution	Reference
**Vaccine coverage**	0.6	0.4−1.0	Triangular (0.4, 0.60, 1.0)	Assumed
**Duration of protection (years)**				(39, 40) and assumed
One-dose	9.0	3.4−14.5	Triangular (3.4, 9.0, 14.5)	…
Two-dose	22.0	19.3−26.2	Triangular (19.3, 22.0, 26.2)	…
Three-dose	44.0	41.0−47.5	Triangular (41.0, 44.0, 47.5)	…
**Vaccine efficacy**				
Against HPV6 and 11 infection				(7, 36)
—HPV-4	0.896	0.793−0.954	Triangular (0.793, 0.896, 0.954)	…
—HPV-9	0.896	0.793−0.954	Triangular (0.793, 0.896, 0.954)	…
Against HPV-16 and 18 infection				(36–38)
—HPV-2	0.829	0.538−0.951	Triangular (0.538, 0.829, 0.951)	…
—HPV-4	0.862	0.694−0.947	Triangular (0.694, 0.862, 0.947)	…
—HPV-9	0.862	0.694−0.947	Triangular (0.694, 0.862, 0.947)	…
Against HPV-31, 33, 45, 52 and 58 infection				(36) and assumed
—HPV-9	0.851	0.837−0.862	Triangular (0.837, 0.851, 0.862)	…
**Efficacy of LEEP**	0.92	0.85−0.99	Beta (50.49, 4.45)	
**Compliance of treatment**				(40, 46–48)
CIN2+	0.95	0.80−1.0	Triangular (0.80, 0.90, 1.0)	…
Genital warts	0.75	0.50−0.80	Triangular (0.50, 0.75, 0.80)	…
Proportion of hysterectomies in CIN3	0.20	0.10−0.50	Triangular (0.1, 0.2, 0.5)	…
**Costs, $**				
**Vaccination**				(49, 50)
—HPV-2 (per dose)	54.4	40.8−54.4	Triangular (40.8, 54.4, 54.4)	…
—HPV-4 (per dose)	127.1	95.3−127.1	Triangular (95.3, 127.1, 127.1)	…
—HPV-9 (per dose)	204.6	153.5−204.6	Triangular (153.5, 204.6, 204.6)	…
VIA	2.8	2.1−3.4	Triangular (2.1, 2.8, 3.4)	(13)
Pap	7.3	6.5−8.2	Triangular (6.5, 7.3, 8.2)	(13)
Colposcopy	6.5	1.9−11.2	Triangular (1.9, 6.5, 11.2)	(13)
Biopsy	18.1	12.2−24.1	Triangular (12.2, 18.1, 24.1)	(13)
LEEP	136.6	102.2−200.8	Triangular(102.2, 136.6, 200.8)	(13)
Hysterectomy	349.0	167.3−530.6	Triangular(167.3, 349.0, 530.6)	(13)
**Cervical cancer and genital warts treatment costs ($)**				(41, 42)
Genital warts	773.8	386.9−876.2	Triangular (386.9, 773.8, 876.2)	…
Local cancer	4754.2	2263.7−7243.5	Triangular (2263.7, 4754.2, 7243.5)	…
Regional cancer	5993.9	1953.0−10033.7	Triangular (1953.0, 5993.9, 10033.7)	…
Distant cancer	7935.8	5164.5−10707.2	Triangular (5164.5, 7935.8, 10707.2)	…
**Annual health-care costs ($)**				(43)
Local cancer	700.0	349.5−1049.5	Triangular (349.5, 700.0, 1049.5)	…
Regional cancer	882.5	441.2−1323.7	Triangular (441.2, 882.5, 1323.7)	…
Distant cancer	1167.8	583.9−1752.8	Triangular (583.9, 1167.8, 1752.8)	…
**Utility score**				(44, 45)
Genital warts	0.83	0.70–0.94	Triangular (0.70, 0.83, 0.94)	…
CIN2 after LEEP	0.98	0.9−1.0	Beta (183.64, 3.75)	…
Post-hysterectomy	0.85	0.82−0.88	Triangular (0.82, 0.85, 0.88)	…
Local cancer	0.83	0.79−0.87	Beta (280.36, 57.42)	…
Regional cancer	0.72	0.65−0.78	Beta (131.26, 51.05)	…
Distant cancer	0.60	0.43−0.77	Beta (18.54, 12.36)	…
Cancer survivor	0.87	0.70−0.99	Triangular (0.70, 0.87, 0.99)	…
**Discount rate**	0.05	0.0−0.08		China Guidelines for Pharmacoeconomic Evaluations

HPV, human papillomavirus; HPV-2, HPV bivalent vaccine; HPV-4, HPV quadrivalent vaccine; HPV-9, HPV nonavalent vaccine; CIN, cervical intraepithelial neoplasia; VIA, Visual inspection with acetic acid; Pap, Papanicolaou test; LEEP, loop electrosurgical excision procedure.

### 2.4 Cost-effectiveness analysis

We assumed a discount rate of 5% for the cost and quality-adjusted life-years (QALYs). We analyzed the results by comparing the incremental cost-effectiveness ratios (ICER). As China does not currently have a specific willingness-to-pay (WTP), we compared the ICER to 1 and 3 times GDP per capita to determine the cost-effectiveness of the program (in 2021, China’s GDP per capita is $12551.5) (51).

### 2.5 Sensitivity analysis

We determined the effect of each parameter on the results through a one-way sensitivity analysis. By conducting a probabilistic sensitivity analysis through 10,000 simulations, we determined the probability of each strategy being cost-effective.

## 3 Results

Compared to the status quo, nine vaccination strategies would incur US$3.057-33.124 million incremental costs and result in 94-1211 incremental QALYs for the 100,000 women cohort (**Supplementary Table 3**). When the same dose of HPV-2, HPV-4, and HPV-9 was administered, HPV-2 vaccination incurred the lowest incremental QALYs. HPV-4 vaccination would result in higher incremental costs as well as incremental QALYs than HPV-2. HPV-9 vaccination increased QALYs marginally compared with 4-valent, but the cost was much higher. When comparing various doses of vaccination, for the same vaccine, the incremental cost and incremental QALYs resulting from three-dose vaccination were greater than two-dose vaccination, and two-dose vaccination is greater than one-dose vaccination.

**Figure 2:**
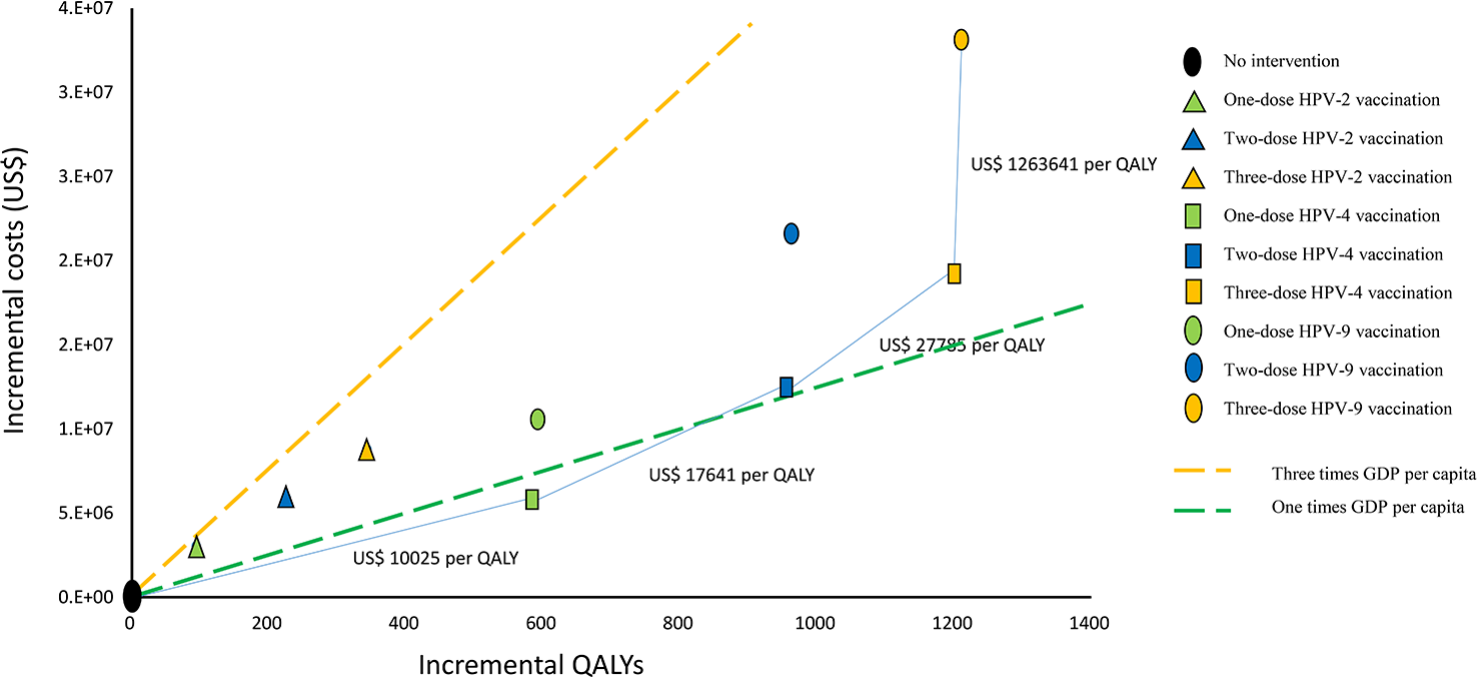
Cost-effectiveness frontier for nine vaccination strategies (100000 cohort members). QALYs, quality-adjusted life years, GDP, gross domestic product, HPV-2: HPV bivalent vaccine, HPV-4, HPV quadrivalent vaccine, HPV-9, HPV nonavalent vaccine.

We calculated the ICER value according to the incremental cost and incremental QALYs (**Figure 2**). The ICER for one-dose HPV-4 vaccination compared to the status quo is US$ 10,025/QALY gained, and the ICER for two-dose HPV-4 vaccination compared to one-dose HPV-4 vaccination is US$ 17,641/QALY gained. The ICER for three-dose HPV-4 vaccination compared to a two-dose HPV-4 vaccination is US$ 27,785/QALY gained, and the ICER for three-dose HPV-9 vaccination compared to three-dose HPV-4 vaccination is US$ 1,263,641/QALY gained.

**Figure 3:**
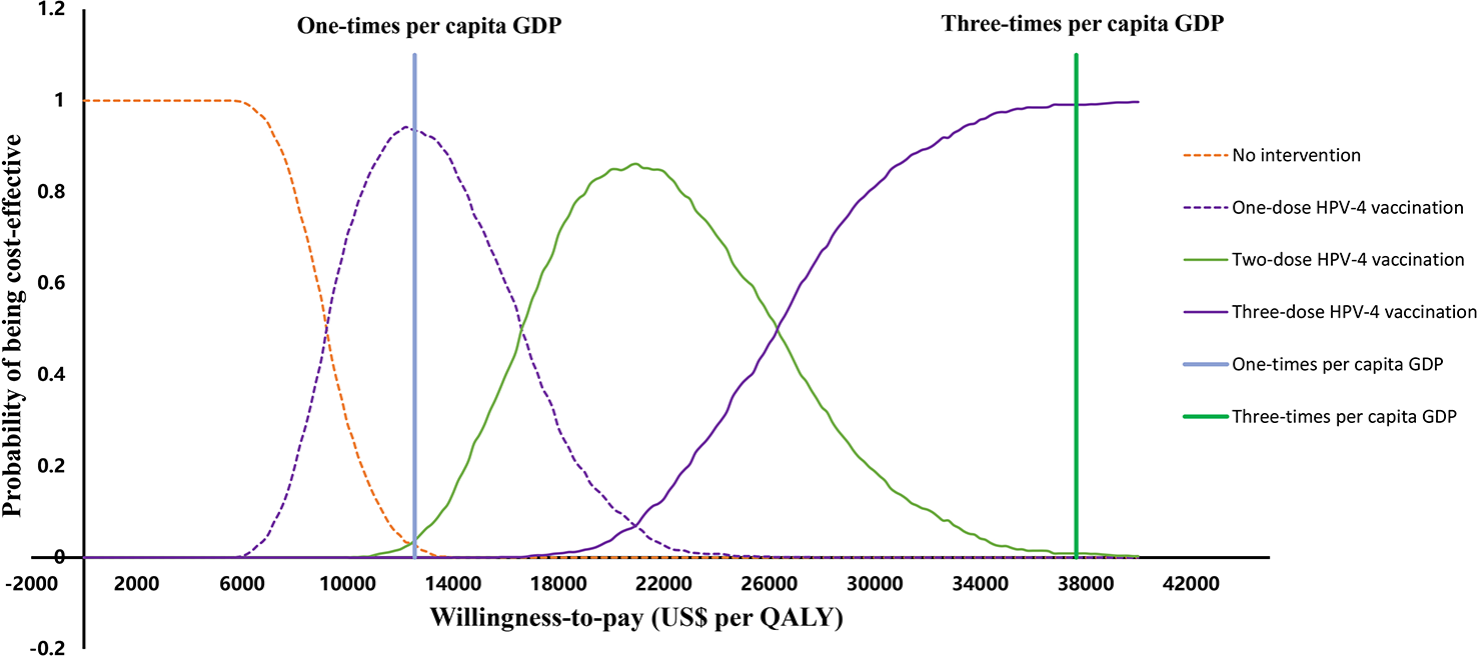
Cost-effectiveness acceptability curves for all strategies. QALYs, quality-adjusted life years, GDP, gross domestic product, HPV-2: HPV bivalent vaccine, HPV-4, HPV quadrivalent vaccine, HPV-9, HPV nonavalent vaccine.

Probability sensitivity analysis illustrates the potential for individual vaccination strategies to be cost-effective when the WTP varies between zero and three times GDP per capita ($37655, **Figure 3**). We found that when the WTP ranged from 0 to $9200, no vaccination was the most cost-effective strategy (100%). When the WTP increased from $9200 to $16600, one-dose HPV-4 vaccination was the most likely to be cost-effective (94.2%); when the WTP increased from $16600 to $26300, two-dose HPV-4 vaccination was the most likely to be cost-effective (86.1%); when the WTP increased to $26300 to $37655 (three times GDP per capita), three-dose HPV-4 vaccination was the most likely to be cost-effective (99.1%).

A univariate sensitivity analysis showed that the cost of the vaccine had a significant impact on the results, and we assumed that the cost of the three vaccines would be 50% and 75% of the current cost respectively. The results show that if the cost of the HPV-4 is reduced to 50% of the current cost, three-dose HPV-4 vaccination is the most cost-effective even at a WTP of three times GDP per capita (**Supplementary Figure 1**). When the cost of imported HPV-9 is reduced to 50% of current costs, when WTP is as low as three times GDP per capita, three-dose HPV-9 vaccination would replace three-dose HPV-4 vaccination as the most cost-effective vaccination strategy (**Supplementary Figure 1**). We found that if the vaccine efficacy of HPV-2, HPV-4, and HPV-9 vaccines all decreases to 70%, at a WTP of three times GDP per capita, two-dose HPV-4 vaccination is the most cost-effective (**Supplementary Figure 2**). Moreover, even if the incidence of infection increases by 20% (**Supplementary Figure 3**), the conclusions on the cost-effectiveness of these vaccination strategies remain unchanged.

## 4 Discussion

Our results showed that for women after treatment for cervical precancerous lesions, the most cost-effective vaccination strategy was a three-dose HPV-4 vaccination when the WTP reached three times GDP per capita. Although the HPV-2 is less expensive than the HPV-4 and HPV-9, the HPV-2 vaccination also produces lower incremental QALYs. While the HPV-9 vaccination demonstrated the largest incremental health benefits, its cost is also the most expensive. The ICER of the HPV-9 vaccination strategy is far greater than the WTP of three times the GDP per capita in China and hence not cost-effective.

The duration of vaccine protection increases with the number of vaccine doses. Three-dose vaccination presented the best protective effect in our model for women after treatment for cervical precancerous lesions. The duration of vaccine protection is an important factor influencing vaccination schedules. However, the implementation of HPV vaccination has only been available for a relatively period worldwide. There is a lack of data from follow-up studies of sufficient duration to explore the duration of HPV vaccine protection. While some studies assume that the protection of HPV vaccines is for life (52), others suggest protection duration varies between one-, two- or three-doses vaccines (16). Currently, reducing vaccine doses may help improve coverage, but the use of single-dose HPV vaccination regimens remains controversial (53). A 2022 randomized control trial showed that single-dose bivalent and nonavalent HPV vaccines were highly effective in preventing oncogenic HPV infection to a level similar to multidose regimens (54). Consistent with this, the WHO Strategic Advisory Group of Experts on Immunization recommends that expanding one- or two-dose vaccination for adolescents 20 years and younger may alleviate the problem of insufficient supply and high costs of vaccines (55). However, further research is still required to inform the effectiveness of single-dose HPV vaccines in older women (27-45 years). In the current context of the global strategy for accelerated eradication of cervical cancer, one- and two-dose vaccination may become increasingly accepted in adolescent women and younger, especially in developing country settings.

Currently, two domestic vaccines and three imported HPV vaccines are on the Chinese market, but all are relatively expensive for an average Chinese family. The high vaccine cost together with the limited supply has resulted in low HPV vaccination coverage in China. With the advance in domestic HPV vaccine development, more domestic HPV vaccines are expected to be marketed in the near future. Further, as Guangdong province has led the way in deploying the country’s first free HPV vaccination programs for school girls (56), the price of HPV vaccines is expected to decline with increased usage and government negotiation. We expect the high price and insufficient supply of vaccines, at least for HPV-2would be mitigated in the foreseeable future, although HPV-4 and HPV-9 may still rely on imports. In our sensitivity analysis, we found that even when the cost of HPV-4 reduces by 50%, the most cost-effective vaccination strategy remains the three-dose HPV-4 vaccination. In contrast, while the cost of HPV-9 reduces by 50%, three-dose HPV-9 vaccination will replace three-dose HPV-4 vaccination as the most cost-effective intervention strategy, at a WTP of three times GDP per capita. HPV-9 has a more comprehensive preventive effect than HPV-4. Several domestic HPV-9 vaccines are currently in clinical trials in China (57), and if successful, a massive reduction in HPV-9 may come into play. However, until this price happens, three-dose HPV-4 vaccination remains the most cost-effective vaccination option for women after treatment for cervical precancerous lesions.

Our study contributes to HPV research by specifically investigating the cost-effectiveness of HPV vaccination among women who had been treated for precancerous cervical lesions. Compared to infection-naïve women, women with a history of HPV infection and cervical lesions may be at a higher risk of HPV re-infection and also less protected by the HPV vaccine (13, 58). With this in mind, we reduced the efficacies of HPV-2, HPV-4, and HPV-9 vaccines in women treated for cervical precancerous lesions to as low as 70% of the corresponding efficacies in HPV infection-naïve women in our sensitivity analysis. Even with this reduction, we found that two-dose HPV-4 vaccination remains the most cost-effective vaccination strategy for women treated for cervical precancerous lesions. Similarly, we also increased the HPV incidence in women treated for precancerous cervical lesions to 20% higher than that of HPV infection-naïve women. This increase did not change the conclusion regarding the cost-effectiveness of two-dose HPV-4 vaccination either. Both sensitivity analyses indicate our conclusion is robust.

Our study also has several limitations. First, we did not consider the cross-protective effects of the vaccines and HPV adverse outcomes outside of precancerous cervical lesions, cervical cancer and genital warts. A recent randomized trial showed that the prophylactic HPV-4 vaccine after treatment has also reduced the occurrence of high-grade CIN (CIN2-3) in the participating women (10), and HPV-2 and HPV-9 vaccines may have similar effects. As our model did not account for these additional health benefits due to the HPV vaccines, our model may underestimate the potential overall health benefits of vaccination. Second, our study focused on adult women with post-treatment cervical precancerous lesions in a Chinese setting and generalization to other country settings may need to be considered with caution. Third, in our model, models for the transmission of low- and high-risk HPV subtypes were simulated independently and did not consider co-infection of low- and high-risk subtypes. Further investigations may be required to validate our conclusions.

In conclusion, our study demonstrated that the three-dose HPV-4 vaccination was the most cost-effective vaccination strategy. However, a substantial reduction in the price of three-dose HPV-9 vaccination may enable it to become the most cost-effective vaccination strategy. Our study provides important evidence for the development of HPV vaccination guidelines and health policies in China.

## Data availability statement

The original contributions presented in the study are included in the article/**Supplementary Material**. Further inquiries can be directed to the corresponding authors.

## Author contributions

MZ designed the project, designed and constructed the model, ran the modeled analyses, interpreted, graphed, and tabulated the results, and was responsible for the write-up of the document. HanL, HuaL and MW contributed to technical and modeling advice throughout the project and critically revised the manuscript. ZZ and LZ supervised all aspects of the study, contributed to the design of the project, the design of the model, and the interpretation of the results, and critically revised the manuscript. All authors contributed to the article and approved the submitted version.
