# Supplementary material for: Vaccinating women previously treated for human papillomavirus-related cervical precancerous lesions is highly cost-effective in China
Source: Front Immunol. 2023 Mar 27;14:1119566. doi: 10.3389/fimmu.2023.1119566 (PMC10083292; doi:10.3389/fimmu.2023.1119566)
Supplement: Supplementary file 1 [file DataSheet_1.pdf]

Supplementary Table 1. Annual transition probabilities of the model

Parameter	Base-case	Range	Distribution	Reference
**HPV infection**				
To HPV(-)				[1; 2; 3; 4]
Low-risk HPV[a]	0.6900	±30%	Uniform (0.4830, 0.8970)	
HPV-16/18[b]				
1 year	0.4002		Uniform (0.2801, 0.5203)	
2 years	0.3917		Uniform (0.2742, 0.5092)	
3 years	0.3388	±30%	Uniform (0.2372, 0.4404)	
4 years	0.3201		Uniform (0.2241, 0.4161)	
≥5 years	0.2111		Uniform (0.1478, 0.2744)	
HPV-31/33/45/52/58[c]				
1 year	0.5987		Uniform (0.4191, 0.7783)	
2 years	0.5422		Uniform (0.3795, 0.7049)	
3 years	0.4869	±30%	Uniform (0.3408, 0.6330)	
4 years	0.2196		Uniform (0.1537, 0.2855)	
≥ 5 years	0.2203		Uniform (0.1542, 0.2864)	
Other high-risk HPV[d]				
1 year	0.5945		Uniform (0.4191, 0.7783)	
2 years	0.4435		Uniform (0.3795, 0.7049)	
3 years	0.4164	±30%	Uniform (0.3408, 0.6330)	
4 years	0.3621		Uniform (0.1537, 0.2855)	
≥ 5 years	0.2059		Uniform (0.1542, 0.2864)	
To genital warts				[5; 6]
Low-risk HPV[a]	0.2850	±30%	Uniform (0.1995, 0.3705)	
To CIN-2 or CIN-3				[7; 8; 9]
HPV-16/18[a]				
1 year	0.0262	±30%	Uniform (0.0183, 0.0341)	
2 years	0.0371		Uniform (0.0260, 0.0482)	

3 years	0.0394		Uniform (0.0276, 0.0512)	
4 years	0.0839		Uniform (0.0587, 0.1091)	
≥ 5 years	0.2144		Uniform (0.1501, 0.2787)	
HPV-31/33/45/52/58[c]				
1 year	0.0058		Uniform (0.0041, 0.0075)	
2 years	0.0192		Uniform (0.0134, 0.0250)	
3 years	0.0395	±30%	Uniform (0.0277, 0.0514)	
4 years	0.0621		Uniform (0.0435, 0.0807)	
≥ 5 years	0.0838		Uniform (0.0587, 0.1089)	
Other high-risk HPV[d]				
1 year	0.0058		Uniform (0.0041, 0.0075)	
2 years	0.0192		Uniform (0.0134, 0.0250)	
3 years	0.0395	±30%	Uniform (0.0277, 0.0514)	
4 years	0.0621		Uniform (0.0435, 0.0807)	
≥ 5 years	0.0838		Uniform (0.0587, 0.1089)	
Proportion of CIN3 in this transition				[7] and assumed
HPV16/18	0.35	0.3-0.5	Triangular (0.3, 0.35, 0.5)	
HPV-31/33/45/52/58[c]	0.2	0.05-0.3	Triangular (0.05, 0.2, 0.3)	
Other high-risk HPV[d]	0.2	0.05-0.3	Triangular (0.05, 0.2, 0.3)	
**Genital warts**				[10; 11] and assumed
To HPV(-) or Low-risk HPV[a]	0.875	0.80-0.90	Uniform (0.80, 0.90)	
Proportion of HPV(-) in this transition	0.79	0.75-0.90	Triangular (0.75, 0.79, 0.90)	…
**CIN-2**				[2; 7; 12] and assumed
To HPV(-) or HPV infection	0.0996	±30%	Uniform (0.0697, 0.1295)	
Proportion of HPV(-) in this transition	0.5	0.4-0.6	Triangular (0.4 0.5 0.6)	

To undetected local cancer				
1−5 years	0.0001		Uniform (0.00007, 0.00013)	
6−10 years	0.0002		Uniform (0.00014, 0.00026)	
11−20 years	0.0029		Uniform (0.00203, 0.00377)	
21−30 years	0.0056		Uniform (0.00392, 0.00728)	
31−34 years	0.0067	±30%	Uniform (0.00469, 0.00871)	
35−39 years	0.0072		Uniform (0.00504, 0.00936)	
40−44 years	0.0155		Uniform (0.01085, 0.02015)	
45−49 years	0.0166		Uniform (0.01162, 0.02158)	
≥ 50 years	0.0177		Uniform (0.01239, 0.02301)	
**CIN-3**				[1; 8; 12] and assumed
To HPV(-) or HPV infection	0.051	±30%		
Proportion of HPV(-) in this transition	0.5	0.4-0.6	Triangular (0.4 0.5 0.6)	
To undetected local cancer				
1−5 years	0.0007		Uniform (0.00049, 0.00091)	
6−10 years	0.0008		Uniform (0.00056, 0.00104)	
11−20 years	0.0142		Uniform (0.00994, 0.01846)	
21−30 years	0.0275		Uniform (0.01925, 0.03575)	
31−34 years	0.0329	±30%	Uniform (0.02303, 0.04277)	
35−39 years	0.0356		Uniform (0.02492, 0.04628)	
40−44 years	0.0753		Uniform (0.05271, 0.09789)	
45−49 years	0.0804		Uniform (0.05628, 0.10452)	
≥ 50 years	0.0855		Uniform (0.05985, 0.11115)	
**Local cancer**				**[1; 2; 8; 11]**
To regional cancer	0.2153	±30%	Uniform (0.1507, 0.2799)	
To death				
1 year	0.024	±30%	Uniform (0.0168, 0.0312)	

2−3 years	0.021		Uniform (0.0147, 0.0273)	
4−20 years	0.02		Uniform (0.0140, 0.0260)	
**Regional cancer**				**[2; 7; 8; 13]**
To distant cancer	0.262	±30%	Uniform (0.1834, 0.3406)	
To death				
1 year	0.127		Uniform (0.0889, 0.1651)	
2−3 years	0.112	±30%	Uniform (0.0784, 0.1456)	
4−20 years	0.059		Uniform (0.0413, 0.0767)	
**Distant cancer**				**[8]**
To death				
1 year	0.3		Uniform (0.2100, 0.3900)	
2−3 years	0.21	±30%	Uniform (0.1470, 0.2730)	
4−20 years	0.087		Uniform (0.0609, 0.1131)	

HPV: human papillomavirus; CIN: cervical intraepithelial neoplasia; LR-HPV(+): HPV6/11 only, no other low-risk HPV infections considered; HPV-16/18(+): infected with HPV-16 alone, with HPV-18 alone or with both HPV-16 and 18; HPV-31/33/45/52/58(+): HPV-31, HPV33, HPV45, HPV52 or HPV58 alone, or co-infection with two or more of these five types of HPV; Other HR-HPV(+): Infection with high-risk HPV types other than HPV16, HPV18, HPV-31, HPV33, HPV45, HPV52 or HPV58.

Supplementary Table 2. Incidence of HPV in specific age groups

HPV type	Age group (years)	Incidence of HPV	Reference
Low-risk HPV[a]	-	0.0261	[14; 15] and assumed
	18-20	0.0270	
	21-24	0.0494	
HPV-16/18[b]	25-29	0.0203	[16]
	30-49	0.0121	
	≥ 50	0.0134	
	18-20	0.0054	
HPV-31/33/45/52/58[c]	21-24	0.0524	[14; 16] and assumed
	25-29	0.0184	

	30-49	0.0343	
	≥ 50	0.0322	
	18-20	0.0034	
	21-24	0.0333	
Other high-risk HPV[d]	25-29	0.0117	
	30-49	0.0218	[14; 16] and assumed
	≥ 50	0.0205	

HPV: human papillomavirus; LR-HPV(+): HPV6/11 only, no other low-risk HPV infections considered; HPV-16/18(+): infected with HPV-16 alone, with HPV-18 alone or with both HPV-16 and 18; HPV-31/33/45/52/58(+): HPV-31, HPV33, HPV45, HPV52 or HPV58 alone, or co-infection with two or more of these five types of HPV; Other HR-HPV(+): Infection with high-risk HPV types other than HPV16, HPV18, HPV-31, HPV33, HPV45, HPV52 or HPV58.

Supplementary Table 3. Incremental costs and incremental QALYs of vaccination strategies versus 'No intervention' (100,000 cohort members)

Vaccination strategy	Costs ($, thousand)	Incremental Costs ($, thousand)	QALYs	Incremental QALYs
No intervention	7506.0	-	1848087	-
One-dose HPV-2 vaccination	10563.0	3056.9	1848181	94
One-dose HPV-4 vaccination	13386.6	5880.6	1848673	587
Two-dose HPV-2 vaccination	13413.9	5907.8	1848312	225
Three-dose HPV-2 vaccination	16306.1	8800.0	1848430	343
One-dose HPV-9 vaccination	18030.1	10524.1	1848677	590
Two-dose HPV-4 vaccination	19862.2	12356.2	1849041	954
Three-dose HPV-4 vaccination	26715.0	19209.0	1849287	1200
Two-dose HPV-9 vaccination	29137.8	21631.8	1849049	962
Three-dose HPV-9 vaccination	40630.4	33124.4	1849298	1211

QALYs: quality-adjusted life years, HPV-2: HPV bivalent vaccine, HPV-4: HPV quadrivalent vaccine, HPV-9: HPV nonavalent vaccine.

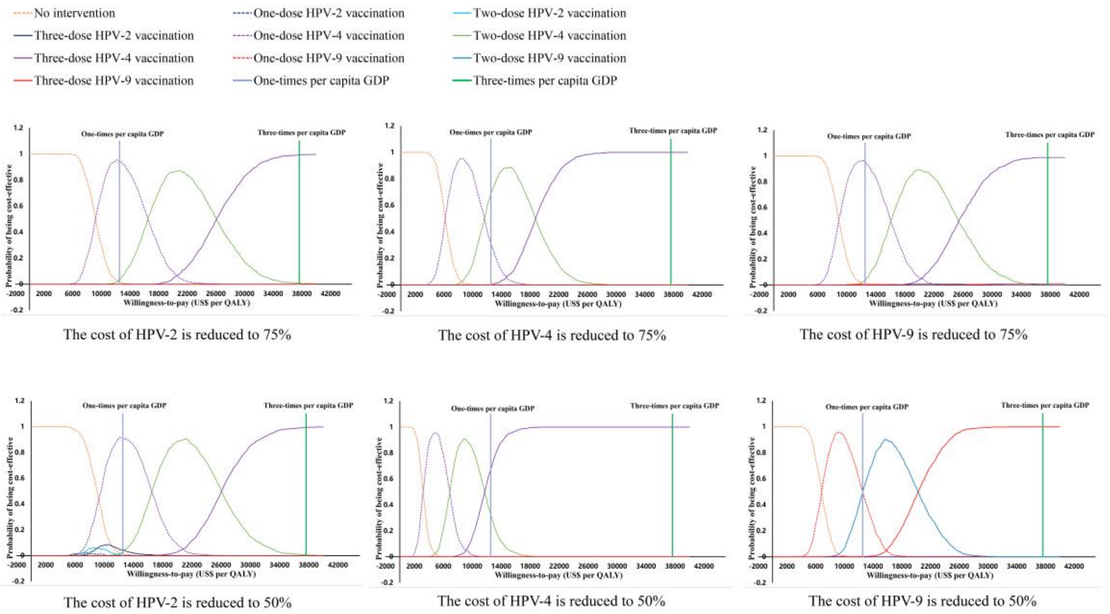

Supplementary Figure 1. Cost-effectiveness acceptability curves for all strategies while the cost of vaccine decreases

QALYs: quality-adjusted life years, GDP: gross domestic product, HPV-2: HPV bivalent vaccine, HPV-4: HPV quadrivalent vaccine, HPV-9: HPV nonavalent vaccine.

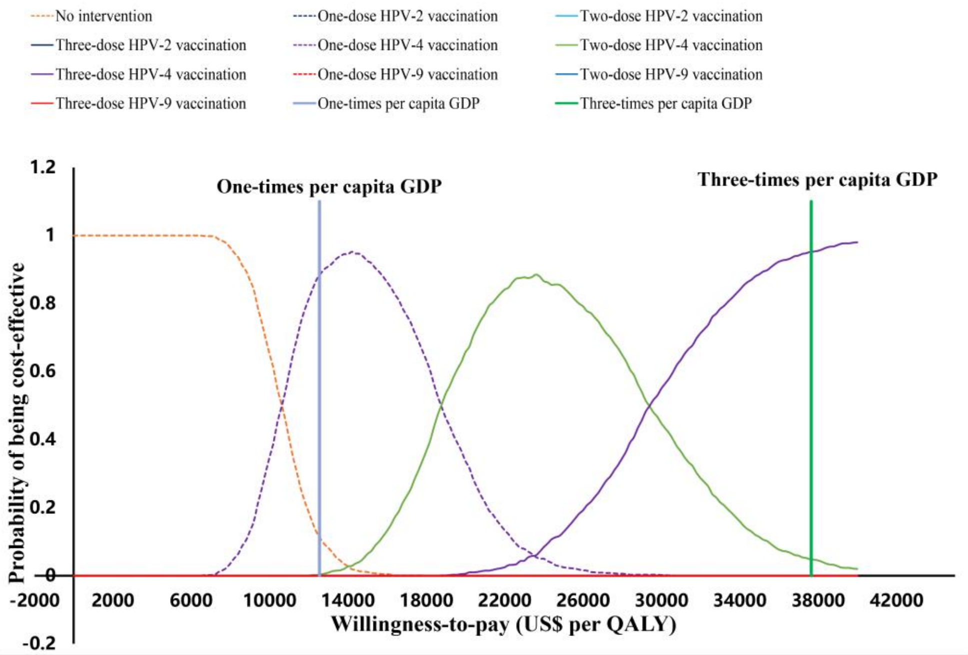

The vaccine efficacy of three types of HPV vaccines decrease to 90%

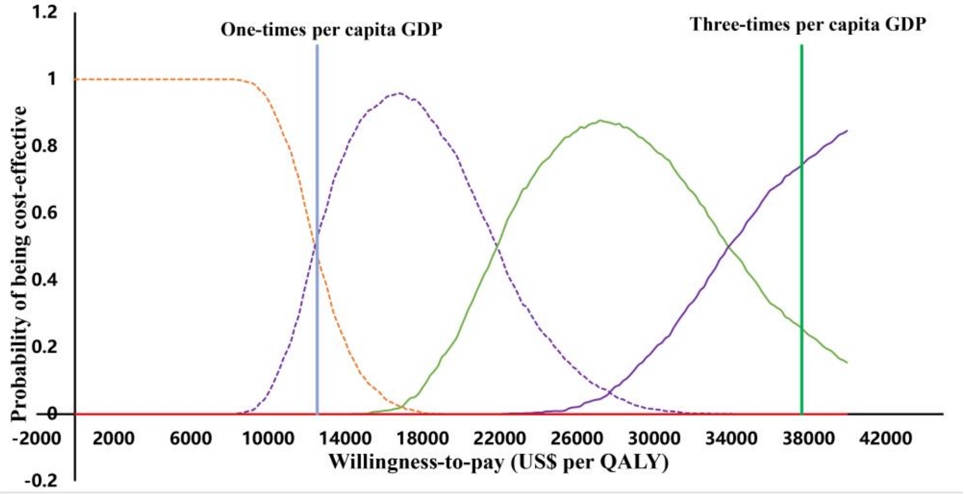

The vaccine efficacy of three types of HPV vaccines decrease to 80%

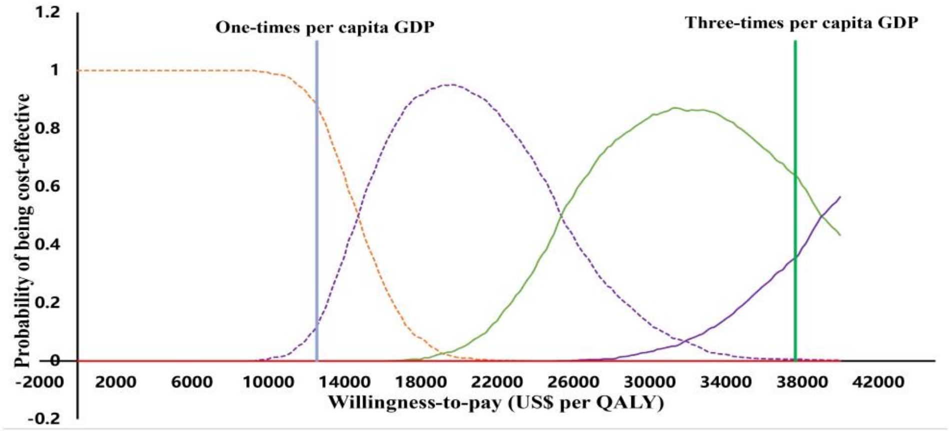

The vaccine efficacy of three types of HPV vaccines decrease to 70%

Supplementary Figure 2. Cost-effectiveness acceptability curves for all strategies (while the vaccine efficacy of three types of HPV vaccines decrease)

QALYs: quality-adjusted life years, GDP: gross domestic product, HPV-2: HPV bivalent vaccine, HPV-4: HPV quadrivalent vaccine, HPV-9: HPV nonavalent vaccine.

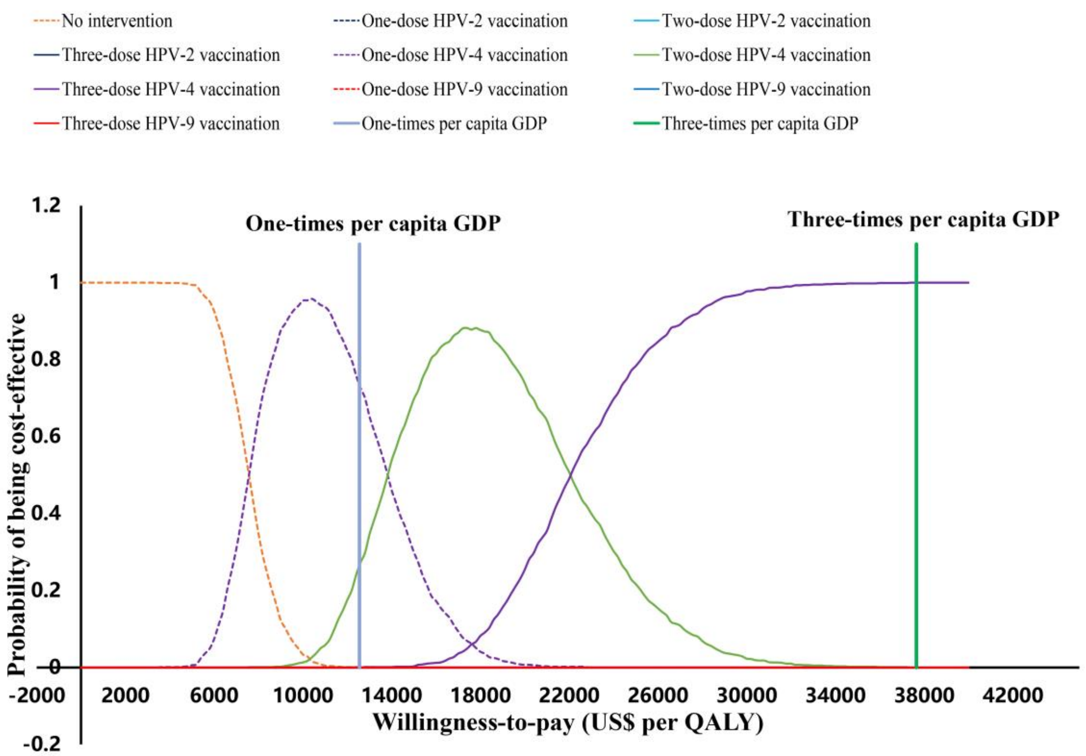

Supplementary Figure 3. Cost-effectiveness acceptability curves for all strategies (Incidence of all types of HPV infection increases by 20%)

QALYs: quality-adjusted life years, GDP: gross domestic product, HPV-2: HPV bivalent vaccine, HPV-4: HPV quadrivalent vaccine, HPV-9: HPV nonavalent vaccine.

[1] D. Yan, Curative effect of laparoscopic hysterectomy in the treatment of early stage cervical cancer and its effect on the prognosis of patients with early stage cervical cancer. CHINA MODERN MEDICINE 23 (2016) 545-55.

[2] J.J. Kim, K.M. Kuntz, N.K. Stout, S. Mahmud, L.L. Villa, E.L. Franco, and S.J. Goldie, Multiparameter calibration of a natural history model of cervical cancer. Am J Epidemiol 166 (2007) 137-50.

[3] C.E. Schmeink, L.F. Massuger, C.H. Lenselink, W.G. Quint, B.I. Witte, J. Berkhof, W.J. Melchers, and R.L. Bekkers, Prospective follow-up of 2,065 young unscreened women to study human papillomavirus incidence and clearance. Int J Cancer 133 (2013) 172-81.

[4] W. Zhang, J. Xiao, and C. Ma, Clearance of high-risk HPV infection in Chinese women with normal cervical cytology. Int J Gynaecol Obstet 118 (2012) 74-5.

[5] R.P. Insinga, E.J. Dasbach, and E.H. Elbasha, Epidemiologic natural history and clinical management of Human Papillomavirus (HPV) Disease: a critical and systematic review of the literature in the development of an HPV dynamic transmission model. BMC Infect Dis 9 (2009) 119.

[6] S.W. Leslie, H. Sajjad, and S. Kumar, Genital Warts, StatPearls, StatPearls Publishing

Copyright © 2021, StatPearls Publishing LLC., Treasure Island (FL), 2021.
[7] N.G. Campos, E.A. Burger, S. Sy, M. Sharma, M. Schiffman, A.C. Rodriguez, A. Hildesheim, R. Herrero, and J.J. Kim, An updated natural history model of cervical cancer: derivation of model parameters. Am J Epidemiol 180 (2014) 545-55.
[8] W. Jing., X. Ke-kui., and S. Bai-gao., An Analysis of the Prognostic Factors in 4374 Cases with Cervical Cancer. China Cancer 23 (2014) 281-87.
[9] Y.L. Guo, K. You, J. Qiao, Y.M. Zhao, C.R. Liu, and L. Geng, Natural history of infections with high-risk HPV in Chinese women with abnormal cervical cytology findings at baseline. Int J Gynaecol Obstet 110 (2010) 137-40.
[10] X. Mo, R.G. Tobe, L. Wang, X. Liu, B. Wu, H. Luo, C. Nagata, R. Mori, and T. Nakayama, Cost-effectiveness analysis of different types of human papillomavirus vaccination combined with a cervical cancer screening program in mainland China. Bmc Infectious Diseases 17 (2017).
[11] D. Yishan., and X. Peizhen., Effect of different surgical approaches on the prognosis of patients with early cervical cancer. Jonrnal of Clinical Medicine in Practice 16 (2012) 30-32.
[12] E.R. Myers, D.C. McCrory, K. Nanda, L. Bastian, and D.B. Matchar, Mathematical model for the natural history of human papillomavirus infection and cervical carcinogenesis. Am J Epidemiol 151 (2000) 1158-71.
[13] L. Sun, X. Sheng, J. Jiang, X. Li, N. Liu, Y. Liu, T. Zhang, D. Li, X. Zhang, and P. Wei, Surgical morbidity and oncologic results after concurrent chemoradiation therapy for advanced cervical cancer. Int J Gynaecol Obstet 125 (2014) 111-5.
[14] L.-P. Luo, P. He, Q.-T. Liu, Y.-H. Jiang, Y.-N. Zhang, Q.-Z. Li, Q. Li, S.-T. Li, F. Yang, H. Ling, X.-G. Dai, Z.-Y. Li, and H.-L. Chen, Prevalence and genotype distribution of HPV infection among 214,715 women from Southern China, 2012-2018: baseline measures prior to mass HPV vaccination. BMC infectious diseases 21 (2021) 328-328.
[15] X. Mo, R. Gai Tobe, L. Wang, X. Liu, B. Wu, H. Luo, C. Nagata, R. Mori, and T. Nakayama, Cost-effectiveness analysis of different types of human papillomavirus vaccination combined with a cervical cancer screening program in mainland China. BMC Infect Dis 17 (2017) 502.
[16] Z. Zou, C.K. Fairley, J.J. Ong, J. Hocking, K. Canfell, X. Ma, E.P.F. Chow, X. Xu, L. Zhang, and G. Zhuang, Domestic HPV vaccine price and economic returns for cervical cancer prevention in China: a cost-effectiveness analysis. Lancet Glob Health 8 (2020) e1335-e1344.

Consolidated Health Economic Evaluation Reporting Standards 2022 (CHEERS 2022) checklist
The CHEERS 2022 statement is intended to be used for any form of health economic evaluation, the new CHEERS checklist contains 28 items with accompanying descriptions.
https://link.springer.com/article/10.1007/s40258-021-00704-x/tables/1

Section/topic	Item No	Guidance for reporting	Reported on page No/line No
**Title**			
Title	1	Identify the study as an economic evaluation and specify the interventions being compared.	1
Abstract			
Abstract	2	Provide a structured summary that highlights context, key methods, results, and alternative analyses.	2
**Introduction**			
Background and objectives	3	Give the context for the study, the study question, and its practical relevance for decision making in policy or	3-4

		practice.	
**Methods**			
Health economic analysis plan	4	Indicate whether a health economic analysis plan was developed and where available.	5
Study population	5	Describe characteristics of the study population (such as age range, demographics, socioeconomic, or clinical characteristics).	5
Setting and location	6	Provide relevant contextual information that may influence findings.	NA
Comparators	7	Describe the interventions or strategies being compared and why chosen.	5
Perspective	8	State the perspective(s) adopted by the study and why chosen.	5
Time horizon	9	State the time horizon for the study and why appropriate.	5
Discount rate	10	Report the discount rate(s) and reason chosen.	6
Selection of outcomes	11	Describe what outcomes were used as the measure(s) of benefit(s) and harm(s).	6
Measurement of outcomes	12	Describe how outcomes used to capture benefit(s) and harm(s) were measured.	6
Valuation of outcomes	13	Describe the population and methods used to measure and value outcomes.	5-6
Measurement and valuation of resources and costs	14	Describe how costs were valued.	5-6
Currency, price date, and conversion	15	Report the dates of the estimated resource quantities and unit costs, plus the currency and year of conversion.	6
Rationale and description of model	16	If modelling is used, describe in detail and why used. Report if the model is publicly available and where it can be accessed.	5
Analytics and assumptions	17	Describe any methods for analysing or statistically transforming data, any extrapolation methods, and approaches for validating any model used.	6
Characterising heterogeneity	18	Describe any methods used for estimating how the results of the study vary for subgroups.	NA
Characterising distributional effects	19	Describe how impacts are distributed across different individuals or adjustments made to reflect priority populations.	NA
Characterising uncertainty	20	Describe methods to characterise any sources of uncertainty in the analysis.	NA
Approach to engagement with patients and others	21	Describe any approaches to engage patients or service recipients, the general public, communities, or stakeholders (such as clinicians or payers) in the design of the study.	NA

affected by the study			
**Results**			
Study parameters	22	Report all analytic inputs (such as values, ranges, references) including uncertainty or distributional assumptions.	5-6<br>Table 1<br>Table S1-3
Summary of main results	23	Report the mean values for the main categories of costs and outcomes of interest and summarise them in the most appropriate overall measure.	7
Effect of uncertainty	24	Describe how uncertainty about analytic judgments, inputs, or projections affect findings. Report the effect of choice of discount rate and time horizon, if applicable.	7
Effect of engagement with patients and others affected by the study	25	Report on any difference patient/service recipient, general public, community, or stakeholder involvement made to the approach or findings of the study.	NA
**Discussion**			
Study findings, limitations, generalisability, and current knowledge	26	Report key findings, limitations, ethical or equity considerations not captured, and how these could affect patients, policy, or practice.	8-9
**Other relevant information**			
Source of funding	27	Describe how the study was funded and any role of the funder in the identification, design, conduct, and reporting of the analysis.	9
Conflicts of interest	28	Report authors conflicts of interest according to journal or International Committee of Medical Journal Editors requirements.	9

D. Husereau, M. Drummond, F. Augustovski, E. de Bekker-Grob, A.H. Briggs, C. Carswell, L. Caulley, N. Chaiyakunapruk, D. Greenberg, E. Loder, J. Mauskopf, C.D. Mullins, S. Petrou, R.-F. Pwu, and S. Staniszewska, Consolidated Health Economic Evaluation Reporting Standards 2022 (CHEERS 2022) Statement: Updated Reporting Guidance for Health Economic Evaluations. Applied Health Economics and Health Policy 20 (2022) 213-221.
